# Effects of alpha-(1,2)-fucosyltransferase genotype variants on plasma metabolome, immune responses and gastrointestinal bacterial enumeration of pigs pre- and post-weaning

**DOI:** 10.1371/journal.pone.0202970

**Published:** 2018-08-27

**Authors:** Ann-Sofie Riis Poulsen, Diana Luise, Mihai Victor Curtasu, Sugiharto Sugiharto, Nuria Canibe, Paolo Trevisi, Charlotte Lauridsen

**Affiliations:** 1 Aarhus University, Faculty of Science and Technology, Department of Animal Science, Tjele, Denmark; 2 Department of Agricultural and Food Sciences (DISTAL), Alma Mater Studiorum—University of Bologna, Bologna, Italy; 3 Diponegoro University, Faculty of Animal and Agricultural Sciences, Semarang, Central Java, Indonesia; National Cancer Institute, UNITED STATES

## Abstract

In pigs, the alpha-(1,2) fucosyltransferase (*FUT1*) gene has been highlighted for its properties in controlling the intestinal expression of enterotoxigenic *E*. *coli* (ETEC) F18 receptors; a pathogen causing edema disease and post-weaning diarrhoea. In this study, we hypothesized that pigs with different genotypes (ETEC F18 resistant (*FUT1*^AA^) versus susceptible (*FUT1*^AG^)) differed in following systemic and enteric responses: growth performance, plasma metabolic profiles, expression of candidate genes for intestinal mucosal homeostasis and immunity, number of selected bacteria and the concentration of short-chain fatty acids (SCFA) in faeces and digesta in piglets pre and post-weaning, and on the ETEC F18 adherence ex vivo. Genotype had the strongest impact on plasma metabolomic profile on day 7 and 28 of age. *FUT1*^AG^ piglets had higher level of N-methyl-2-pyrrolidinone, hippuric acid, oxindole, and 3-oxo-5-beta-chol-7-en-24-oic acid on day 7, and a higher level of guanosine on day 28 than that in the *FUT1*^AA^ piglets. *FUT1*^AA^ piglets had a higher level of betaine on day 7 and 3-methylguanine on day 28. On day 34 of age, the *FUT1*^AA^ pigs had higher levels of S-2-hydroxyglutarate, L-phenylalanine, tauroursodeoxycholic acid and an undetermined PC/LysoPC, while Ile Glu Phe Gly peptide and genistein 5-O-glucuronide, and PC (18:0/0:0) were at higher levels in the *FUT1*^AG^ piglets. *FUT1* genotype did not affect the growth performance and expression of candidate genes. *FUT1*^AG^ piglets had a higher number of haemolytic bacteria in faeces and in digesta than that in *FUT1*^AA^ at 34 days of age. The colonic acetic acid concentration was highest in *FUT1*^AG^ piglets. *FUT1* genotype may influence not only the expression of *E*. *coli* F18 receptors but could potentially impact the gut homeostasis and metabotype of piglets pre and post-weaning. Further investigations on the relation between *FUT1* genotype and these aspects including the intestinal commensal microbiota will expand the knowledge on factors affecting the intestinal ecosystem.

## Introduction

The gut microbiota composition and host genetics background are suggested to play key roles in the development of inflammatory intestinal diseases [1]. In pigs, the alpha-(1,2) fucosyltransferase (*FUT1*) gene has been highlighted for its properties in controlling the intestinal expression of enterotoxigenic *E*. *coli* (ETEC) F18 receptors; a pathogen causing edema disease and post-weaning diarrhoea. In humans, the fucosyltransferase 2 (*FUT2)* gene is responsible for H-antigen expression in the intestine, and this structure has been shown to be particularly relevant in pathogen adhesion and for the susceptibility to chronic inflammation in the intestine, e.g., inflammatory bowel disease (IBD), Crohn’s disease (CD) and ulcerative colitis (UC) [2–3]. Indeed, genome wide associate studies have associated the mutation in the *FUT2* gene to the risk of developing CD and UC [2–3]. In pigs, both the *FUT1* and *FUT2* genes are expressed in the intestines and the *FUT1* is considered to be orthologous to the human H gene which catalyses the addition of fucose to a terminal galactose in an alpha 1,2-linkage [4].

Sophisticated genetic tools have extensively been used to genetically select pigs with favourable production traits such as increased growth rate, carcass weight, number of piglets per litter, and improved meat quality, while less focus has been given to animal health and robustness. However, the growing concern regarding bacterial antibiotic resistance has increased the demand for alternative therapeutic or preventive options when dealing with infectious diseases. Molecular genetics enables development of pig selection schemes that can include genetic markers associated with innate disease resistance. *Escherichia coli (E*. *coli)* F18 is a highly pathogenic bacterium causing post-weaning diarrhoea (PWD) and edema disease (ED) in young pigs, and until now, successful methods to control this bacterium remains to be developed [4,5]. Breeding F18-resistant piglets has been studied as a potential preventative strategy to limit PWD and ED [6]. *E*. *coli* F18 recognizes and adheres to highly specific receptors located on the small intestinal brush border. Previous studies have suggested that the F18 receptor has a glycophospholipid structure and that its expression is genetically controlled by *FUT1* gene located near the blood group inhibitor S on chromosome 6 [7,8]. Meijerink and co-authors [9] proposed that the single point mutation G>A at base-pair 307 in the *FUT1* gene influences piglet susceptibility to *E*. *coli* F18 diarrhoea, classifying piglets with the *FUT1*^AA^ genotype as resistant animals and piglets with the *FUT1*^AG^ and *FUT1*^GG^ genotypes as susceptible. The *FUT1*^AA^ genotype leads to a lower level of *FUT1* and *FUT2* genes expression, which encode for galactoside 2-L-fucosyltransferase enzyme (FUT2), and thus reduce the quantity of this enzyme [4]. In humans, the FUT2 enzyme catalyses the addition of terminal alpha (1,2) fucose residues, producing the H type 1 carbohydrate expressed on the surface of epithelial cells and in mucosal secretions of secretor individuals [10]. The fucosylation of carbohydrate structures expressed on cell surfaces of the gut by glycoproteins, glycolipids, and proteoglycans have been associated with biological processes such as inflammation, host–pathogen interactions, and tumor metastasis [11,12]. A recent study by Hesselager et al. (2016) showed that there are significant differences in the O-linked glycans of the intestinal mucosal proteins between *FUT1*^AA^ and *FUT1*^AG^ pigs [13]. The *FUT1*^AG^ lead a higher level of O-glycan structures that were fucosylated to show H-antigens [13]. Accordingly, a previous study in mice [14] showed that the intestinal alpha (1,2) fucosylation in *FUT2*-null mice, which are characterized by a null expression of *FUT2* gene and were obtained by replacing *FUT2* with the bacterial reporter gene lacZ [15], had a lower level of fucosylation in the intestinal mucosa. Additional studies in mice showed that the intestinal alpha-(1,2) fucosylation represents a significant energy source for some bacteria, suggesting that alpha-(1,2) fucosylated glycans may contribute to the establishment and maintenance of the commensal microbial community [16].

Nevertheless, so far there exists only limited knowledge of *FUT1* polymorphism and pig-gut microbiota interactions. Both, genotype and the gut microbiota, are reported to be intrinsic contributors in regulating the gut mucosal maturation, the intestinal changes occurring during the weaning transition, and more generally, the overall health of the host [17–19]. In the present study we, therefore, developed the hypothesis that *FUT1* genotype (*E*. *coli* F18 susceptible versus resistant) would affect the host metabolism, immune response, and the gastrointestinal microbiota composition in pigs during the suckling and post-weaning period. The dual purposes of the study were to characterise the metabolomic profile and immunological parameters of the gut epithelium of *FUT1*^AA^ and *FUT1*^AG^ piglets and to investigate how *FUT1* genotype influences the establishment of the gastrointestinal microbiota, using classical culture techniques, in 5 to 34 days old piglets.

## Methods

The experiment was carried out at the experimental facility at the Department of Animal Science (Foulum, Aarhus University). Animals were reared following Danish guidelines for animal care and use. The protocol was approved by Danish Animal Experiments Inspectorate, Ministry of Food, Agriculture and Fisheries, Danish Veterinary and Food Administration (Protocol Number: 2012-15-2934-00125).

### Animals, study design, and *FUT1* genotyping

A total of 17 piglets were included in the study. The animals were obtained by breeding two *FUT1*^AG^ sows with the same *FUT1*^AA^ boar ((Danish Landrace x Yorkshire breed) x Duroc), providing *FUT*^AG^ and *FUT1*^AA^, but no *FUT1*^GG^, piglets. *FUT1*^AG^ and *FUT1*^GG^ piglets are considered equally susceptible to enterotoxigenic *Escherichia coli* F18 [5]. An ear tissue sample was collected shortly after birth and used for *FUT1* genotyping. In brief, the genotyping procedure included a PCR amplification of the *FUT1* gene product harbouring the mutation of interest. Subsequently, the PCR fragments were digested with the HinP1I restriction enzyme and run on a 1.5% agarose gel according to Bao et al. [20]. Genetic testing revealed that ten piglets were of the sensitive genotype *FUT1*^AG^ and seven piglets were of the resistant genotype *FUT1*^AA.^ (3 *FUT1*^AG^ and 3 *FUT1*^GG^ in one litter; 7 *FUT1*^AG^ and 4 *FUT1*^GG^ in the other litter).

The piglets were raised with their dam until the day of weaning (day 28), and had free access to water and creep feed (without added zinc oxide) from day 21 of age. After weaning, piglets were housed together in pens according to litter and fed a standard post-weaning diet (without added zinc oxide). The temperature in the weaning unit was 23°C and piglets had free access to rooting material. All animals were individually weighed once a week from day 7 of age, and the average daily weight gain (ADWG) was calculated. On day 34 after birth animals were sacrificed by a trained staff using a penetrating captive bolt followed by exsanguination at our facility, to avoid stress due to transportation.

### Sample collection

Blood samples were collected in EDTA-containing vacutainers (Vacuette, Greiner Bio-One GmbH, Kremsmünster, Austria) from the jugular vein on day 7, 28, and 34 after birth. The blood samples were immediately centrifuged at 3,000 × *g* for 10 min, and the plasma was stored at –80°C for further analyses of the metabolic profile. Faecal samples were collected directly from the rectum at 5, 7, 14, 21, 28, and 34 days of age. Subsamples were stored at -20°C for organic acid analysis. Bacterial enumerations by plating were performed on a subsample of fresh faeces. Due to the small size of the piglets, adequate quantities of faeces for microbial analyses and of blood for metabolomics analysis could not be sampled from all piglets at all ages.

All piglets were sacrificed at 34 days of age. The abdomen was incised and the gastrointestinal tract removed. Luminal contents (digesta) from the stomach, last third of distal small intestine, caecum, and middle part of the colon were sampled immediately and subsamples were stored at -20°C for organic acid analysis. Bacterial enumerations by plating were performed on fresh subsamples. After removal of the lumen content, the epithelium was rinsed and mucosal samples were obtained from four gastrointestinal segments (stomach, ileum, caecum, and mid colon). The samples were stored at -80°C for further candidate gene expression analyses. In addition, 15 cm of distal small intestine were collected in order to investigate the adherence of *E*. *coli* O138:F18 to the mucosa using the Porcine Intestinal Organ Culture (PIOC) procedure [21].

### LC-MS metabolomics determination

The LC-MS metabolomic profile of the plasma samples was analysed using a Dionex UltiMate 3000 (Dionex, Sunnyvale, CA, USA) ultra-performance liquid chromatography (UPLC) system, coupled to a MaXis impact Quadrupole Time-of-Flight (QTOF) mass spectrometer (Bruker Daltonics GmbH, Bremen, Germany) operating in positive and negative electrospray ionization mode (ESI^+^, ESI^-^). The analytical column was an Acquity UPLC HSS T3 (2.1 x 100 mm, 1.8 μm; Waters, Milford, MA, USA), kept at 30°C. Sample preparation and the LC-MS analysis were performed as described by Ingerslev et al. [22]. In order to evaluate the performance of the analytical system, blank samples (100% acetonitrile) and pooled plasma samples from the pigs at each time point were used as quality controls and reinjected for every six samples.

Data acquisition and control of UPLC/MS was done using oTOF control v.3.4 and HyStar v.3.2 (Bruker Daltonics GmbH, Bremen, Germany). Data pre-processing was performed by calibrating the mass spectrum using Data Analysis v.4.0 (Bruker Daltonics GmbH, Bremen, Germany) followed by the Find Molecular Features (FMF) tool. Parameters for compound detection with FMF were set to 5 for S/N threshold, 8 spectra for compound length, and a smoothing width of 2 for the peaks. Other alterations of the data included shortening of the chromatogram retention times to exclude noise and carry-over compounds, the mass range was kept between m/z 50 and 1000, and the most common adducts in positive and negative mode were subtracted.

### Metabolite identification

Metabolites resulting statistically different between the experimental groups were identified using METLIN (http://masspec.scripps.edu/), the Human Metabolome Database (HMDB, http://www.hmdb.ca/), and LIPID MAPS database (http://www.lipidmaps.org/) searches. Confirmation of compounds was done by comparison of retention times and MS/MS fragmentation patterns to those found in METLIN or HMDB, or to commercial standard compounds when available. Metabolites were reported as described by Sumner et al. [23] using the four level identification system: 1. Identified compounds; 2. Putatively annotated compounds; 3. Putatively characterized compound classes; 4. Unknown compounds.

### Gene expression analysis

Total RNA was extracted from the samples of the four tissues using the NucleoSpin RNA kit (Macherey-Nagel) according to the manufacturer’s protocols. The quality and quantity of RNA were evaluated using agarose gel electrophoresis and a spectrophotometer (NanoDrop ND-1000; Saveen Werner), respectively. The sample concentration was adjusted to 200 ng/μl, and the RNA then converted into complementary DNA using the High Capacity cDNA Reverse Transcription Kit (Invitrogen) according to the manufacturer’s instructions. The qPCR of glyceraldehyde-3-phosphate dehydrogenase (*GAPDH*), Interleukin 10 (*IL10*); Tumor Necrosis Factor Alpha (*TNF-α*), cyclo-oxygenase-2 (*COX-2*), Zona Occludens 1 (*ZO-1*), and Occludin (*OCLN*) genes were run in 384-well plates by mixing 2 μl of complementary DNA with 8μl of a mix containing 5 pmol of each primer, 1pmol of the probe and 5μl TaqMan Master Mix (catalogue no. 4324018; Applied Biosystems). The sequence (5’-3’) of primers and probes for *GAPDH*, *TNF-α*, *IL10*, and *COX-2* genes are reported in Sugiharto et al. [24], while for the *ZO-1* and *OCLN* genes, the primers and probe from 20 x TaqMan Gene Expression Assay, catalogue no. 4351372 (Applied Biosystems), were used. The analysis was performed in duplicates for the target genes and in triplicates for the housekeeping gene (*GAPDH*) under standard amplification conditions determined for the Applied Biosystem ViiA 7 Real-Time PCR system (Life Technologies). The data were normalized to the expression of the housekeeping gene. Relative quantitation of gene expression was calculated using the 2^-ΔΔ*Ct*^ method.

### Dry matter and organic acid analysis

Dry matter content of digesta was determined by freeze-drying (ScanVac Coolsafe 55, Labogene ApS, Lynge, Denmark). The concentration of short-chain fatty acids and lactic acid in faeces and digesta were measured as previously described by Canibe et al. [25].

### Microbiological enumerations by plating

Faecal samples (approximately 1 g) were transferred to plastic bags and 5 or 10 ml pre-reduced salt medium was added. The content was homogenised in a Smasher paddle blender (bioMérieux Industry, USA) for 2 minutes. Digesta samples (approximately 5 g) were transferred to flasks containing 50 ml pre-reduced salt medium [26]. The flask content was transferred to a CO_2_ flushed bag and homogenized in a stomacher blender for 2 minutes. 1 ml homogenate was transferred to a Hungate tube containing 9 ml pre-reduced salt medium and 10-fold dilutions were prepared using the technique previously described by Miller and Wolin [27]. Each sample was plated on selective (and indicative) and non-selective agar plates.

Enterobacteriaceae were enumerated on MacConkey agar (Merck 1.05465) after aerobic incubation for 1 day. Haemolytic bacteria were enumerated on blood agar (Oxoid Pb5039A) after aerobic incubation for 1 day. *Clostridium perfringens* were enumerated using the pour-plate technique on Tryptose Sulfite Cycloserine agar (Merck 1.11972 and 1.00888) after anaerobic incubation for 1 day. Lactic acid bacteria were enumerated on de Man, Rogosa and Sharp agar (Merck 1.10660) after anaerobic incubation for 2 days. Total anaerobic bacteria were enumerated in roll tubes containing pig colon fluid-glucose-cellobiose agar and incubated for 7 days [26]. All plates and roll-tubes were incubated at 37˚C.

### Porcine Intestinal Organ Culture (PIOC)

The PIOC procedure was included to investigate the adherence of *E*. *coli* O138:F18 to the mucosa of *FUT1*^AA^ and *FUT1*^AG^ piglets following the procedure previously described by Sugiharto et al. [21]. In short, a distal small intestinal segment (15 cm) was sampled and incubated with a solution containing *E*. *coli* O138:F18 for 1 hour. The tissue was then homogenized, diluted, and *E*. *coli* enumerated on MacConkey (Merck 1.05465) agar plates after aerobic incubation at 37°C overnight.

### Statistical analysis

Weekly body weight (BW) and average daily weight gain (ADWG) were analysed using an ANOVA with a factor design including the *FUT1* genotype and litter as fixed effects, and the initial body weight as a covariate.

With regard to the metabolomics data, the initial step encompassed multi-level PCA (MLPCA) carried out on the full dataset (day 7, 28, 34) and separately on the individual time datasets in order to highlight possible sub-structures in the data. The data was Pareto-scaled and potential outliers were removed after initial visual inspection of PCA plots. To evaluate metabolite differences between *FUT1* genotypes, potential confounding factors were evaluated by using a linear regression model. The linear regression model included sex, litter, and weight of the animals at the three different time points (7, 28 and 34) as fixed factors. Confounding effects were removed by computing the residuals of model considering the factors described in the linear regression model. All further analyses were based on these residuals. A sparse Multilevel Partial Least Squares Discriminant Analysis (sMLPLS-DA) [28] was used to investigate differences in the plasma metabolic profile between the two *FUT1* genotypes on each of the three-time point. sMLPLS-DA was applied using a dummy variable distinguishing the two genotype (AA and AG) as a response variable and the scaled subject variations as predictors. This version involves the automatically choice, using an internal 5-fold cross-validation procedure of a sparseness coefficient that controls the trade-off between the goodness-of-fit of the model and its complexity (i.e., the number of selected metabolites).

Furthermore, in order to evaluate both the stability and the effect size of the metabolites contributing with non-null coefficients to discriminant dimensions, a validation test of 1000 permutation test (permutation of predictors) coupled with a Leave One Out (LOO) procedure was applied. Metabolites having a P < 0.1 both for effect size (P_si_) and stability (P_st_), simultaneously, were considered stable and significant.

All statistical analyses on metabolomics data was performed in R v. 3.0.3 (R Development Core Team, 2008) using the “*lme4*” package [29] for the computation of metabolite residuals, the “spls” package [30] (function *cv*.*splsda* and *splsda*) for the sMLPLS-DA analysis and the function “prcomp” for PCA.

The impact of genetic variation *FUT1*^AA^ *versus FUT1*^AG^ on the expression of candidate pro-inflammatory and intestinal barrier regulation genes among the different intestinal tissues was determined using a linear model in which the *FUT1* genotype, intestinal tissue, and their interaction were included as fixed effects while litter was included as random effects, and pigs as a repeated measurement.

The effects of *FUT1* genotype and age on bacterial composition, organic acid concentration, and pH, were analysed by fitting the data to a linear mixed model in which genotype and age/intestinal segment were included as fixed effects, while litter was included as random effect, and pig as repeated measurement (by including random intercept terms) to account for multiple observations within the same litter or within the same pig. The fixed effects were tested using an F-test with Kenward-Roger approximation, where the reduced model was tested against the full model. When age was found to have a significant effect, a post-hoc test was performed, including Bonferroni adjustment to correct for multiple comparisons [31]. Effects were considered significant when P < 0.05, whereas when P > 0.05 but ≤ 0.10 differences were considered to indicate a trend towards a significant effect.

Statistical analysis was performed with R statistical software (R Development Core Team, 2008) using the “*lm”* function of “*stats”* package version 3.3.0 for gene expression and piglet performance data. The “*lmer”* function from the “*lme4”* package [29], the “*KRmodcomp”* function in the “*pbkrtest”* package [31], and the “*multcomp"* package [32] were applied for the pH, bacterial and organic acid parameters.

## Results

All pigs remained healthy during the trial. Analyses of the data according to the described statistical model revealed that genotype had no significant effect on BW and ADWG during the entire period of the trial. The ADWG from day 7 to day 34 of age was 0.196 kg/day for the *FUT1*^*AG*^ and 0.153 kg/day for the *FUT1*^AA^ genotype (SEM = 0.017 kg/day).

### Plasma metabolomics profile

**Fig 1:**
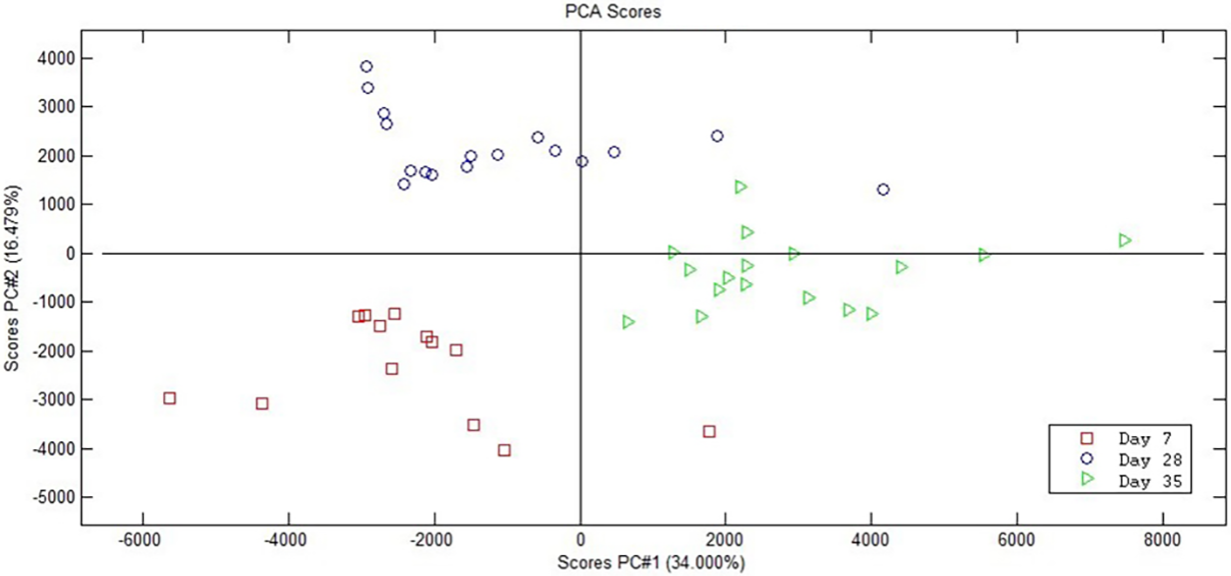
PCA score plot of the piglets plasma metabolomic profiles at 7, 28, and 34 days of age. The variances accounted by the principal components are shown in the axes.

**Fig 2:**
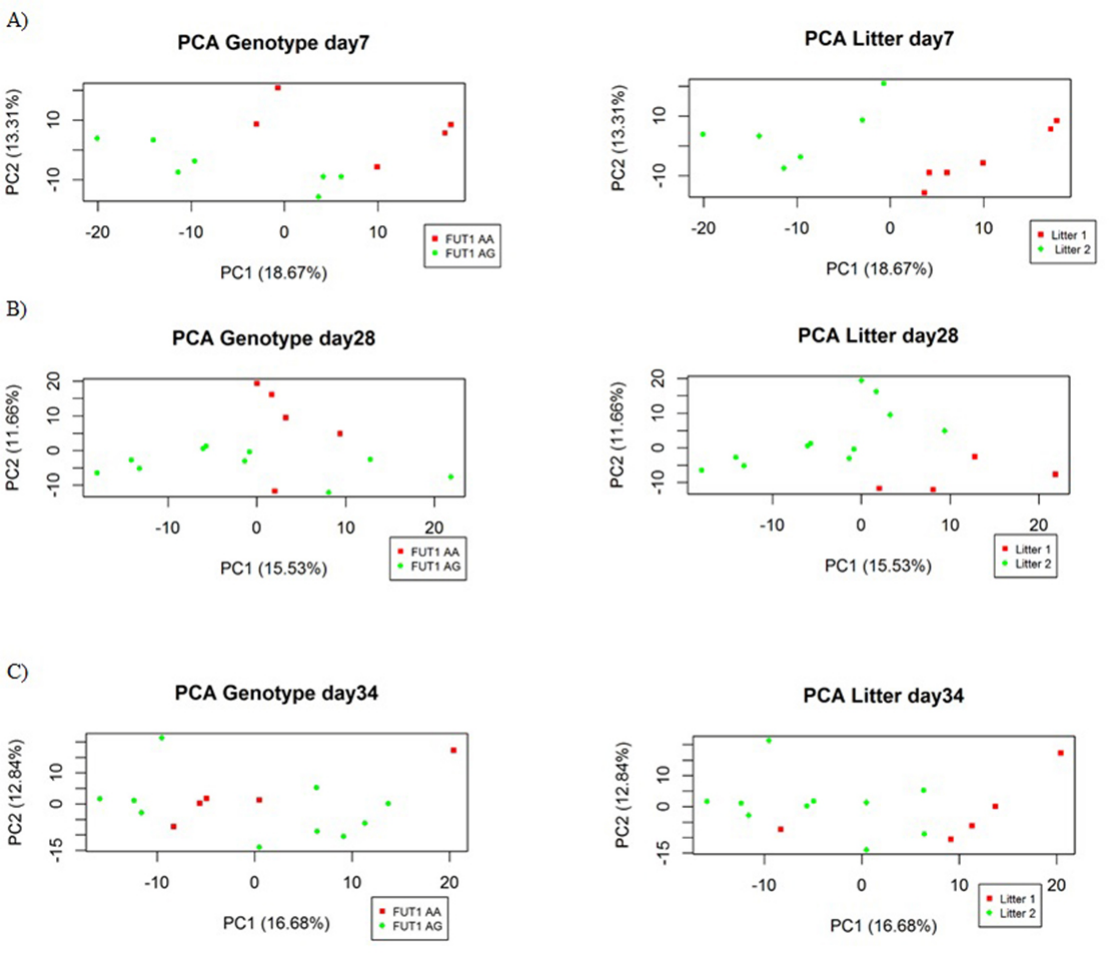
PCA Score plot of *FUT1* genotype and litter differences during suckling, weaning, and after weaning. **A)**: Score plot of plasma metabolites on day 7. PC1 and PC2 explain 18.67% and 13.31%, respectively; **B)** Metabolomics **s**core plot on day 28 (weaning), PC1 = 15.53% and PC2 = 11.66%. **C)** PCA score plot after weaning (day 34), where PC1 and PC2 explain 16.68% and 12.84% of total variance, respectively.

The score plot of the Principal Component Analysis (PCA) conducted on plasma samples is shown in Fig 1. Clusters according to piglet age were clearly visible both during the suckling period (day 7 *versus* day 28) and after weaning (day 28 *versus* day 34). After outlier’s filtration (2 outliers on day 7; 1 outlier on day 28 and day 34), the datasets were composed of 12 pigs (7 *FUT1*^AG^ and 5 *FUT1*^AA^) and 789 metabolites on day 7, and 15 animals (10 *FUT1*^AG^ and 5 *FUT1*^AA^) with 727 metabolites on day 28, and 707 compounds on day 34. The litter and genotype effects in the single time datasets are presented in the PCA score plot (Fig 2). At 7 days of age, the effects of both litter and *FUT1* genotype were evident on the metabolic profile by exploring the first (PC1) and second principal component (PC2), which explained 18.7% and 13.3% of the total variance, respectively (Fig 2A). The Fig 2B shows that *FUT1*^AA^ and *FUT1*^AG^ clustered into two groups along the PC2 (explained 11.7% of the total variance). The PCA showed a clear separation according to litter along the PC1 (explained 15.5% of total variance) on day 28. Litter and genotype did not have an effect on the metabolic profile on day 34. Discriminant identified compounds (P ≤ 0.01) for stability and effect size obtained from sMLPLS-DA coupled with LOO validation model in plasma metabolic profiles of *FUT1*^AG^ and *FUT1*^AA^ animals on day 7, 28, and 34 are listed in **Table 1**, while a complete list, including the unidentified compounds, is shown in S1 Table (**S1 Table**). Considering the discriminant identified compounds on day 7, the plasma metabolic profile of *FUT1*^AG^ pigs was characterised by a higher level of N-Methyl-2-pyrrolidinone (P_si_ = 0.01; P_st_ = 0.01), hippuric acid (P_si_ = 0.01; P_st_ = 0.08), Oxindole (P_si_ = 0.02; P_st_ = 0.07) and 3-Oxo-5-beta-chol-7-en-24-oic Acid (P_si_ = 0.02; P_st_ = 0.08) compared to the profile of *FUT1*^AA^ piglets, which had a higher level of Betaine (P_si_ = 0.03; P_st_ = 0.08) (**Table 1**). At weaning (day 28 of age), two identified compounds discriminated the *FUT1* genotypes; 3-methylguanine (P_si_ <0.001; P_st_ = 0.001) characterised the *FUT1*^AG^, and guanosine (P_si_<0.001; P_st_ = 0.02) the *FUT1*^AA^ piglets. Eight identified compounds discriminated the *FUT1*^AA^ and *FUT1*^AG^ pigs based on their metabolome on day 34 of age. (S)-2-hydroxyglutarate (P_si_ = 0.02; P_st_ = 0.001), L-phenylalanine (P_si_ = 0.04; P_st_ = 0.07), PC 18:0/0:0 (P_si_ = 0.04; P_st_ = 0.03), and tauroursodeoxycholic acid (P_si_ = 0.02; P_st_ = 0.06 positive mode; P_si_ = 0.03; P_st_ = 0.08 negative mode) were higher in plasma of pigs with the *FUT1*^AA^ genotype, while the *FUT1*^AG^ piglets had higher values of an unidentified PC/ LysoPC (P_si_ = 0.03; P_st_ = 0.001), Ile Glu Phe Gly peptide (P_si_ = 0.04; P_st_ = 0.01) and genistein 5-O-glucuronide (Baicalin). (P_si_ = 0.02; P_st_ = 0.07) (**Table 1**).

**Table 1:** Discriminant plasma metabolites for *FUT1*^AA^ and *FUT1*^AG^ obtained from piglets at 7, 28 and 34 days of age by the sMLPLS-DA.

Item		ID level^1^	Charge	Retention time (min)	m/z^2^	Genotype								
						*FUT1*^AG^		*FUT1*^AA^		Stability^3^		Effect size^4^		Direction^5^
						Mean	ds	Mean	ds	t	P	t	P	
*Day 7 of age*														
	[M+Na] Betaine	1	Pos	0.63	140.9	6589	207	6794	195.3	6	0.077	-0.043	0.027	AA
	N-methyl-2-pyrrolidinone	2	Pos	1.91	102.08	8167	160.9	7242	237.3	10	0.011	0.047	0.012	AG
	Hippuric acid	1	Pos	3.26	180.06	13273	2988	4546	1734	6	0.076	0.04	0.014	AG
	Oxindole	2	Pos	3.37	134.06	12923	2446	9476	840.5	6	0.069	0.042	0.015	AG
	3-Oxo-5beta-chol-7-en-24-oic Acid	3	Pos	7.28	374.28	9412	288.2	8140	440	6	0.073	0.043	0.024	AG
*Day 28 of age*														
	3-Methylguanine	2	Pos	0.91	166.07	5824	241.9	7044	552.9	15	0.001	0.462	0	AA
	Guanosine	1	Pos	1.27	284.1	7980	1016	4533	1646	12	0.021	0.417	0	AG
*Day 34 of age*														
	(S)-2-Hydroxyglutarate	2	Neg	1.9	147.05	15301	796.7	17737	1058	15	0.001	-0.06	0.022	AA
	L-Phenylalanine	1	Neg	1.9	164.07	122786	6631	135175	8330	8	0.07	-0.042	0.037	AA
	Tauroursodeoxycholic acid	2	Neg	5.62	498.29	21853	26903	73051	80247	7	0.077	-0.044	0.027	AA
	Tauroursodeoxycholic acid	2	Pos	5.65	500.31	7702	4255	14039	15506	8	0.058	-0.042	0.023	AA
	Ile Glu Phe Gly	3	Pos	3.89	465.23	5903	2108	5588	1010	14	0.008	0.051	0.038	AG
	Genistein 5-O-glucuronide,	3	Neg	4.18	445.07	6445	2269	3886	1323	8	0.071	0.045	0.022	AG
	Unknown PC/LysoPC	3	Pos	8.9	560.3	1436	106.7	1750	196.9	15	0.001	-0.056	0.033	AA
	PC(18:0/0:0)	3	Neg	10.45	560.33	7489	7674	4109	2918	11	0.026	0.046	0.044	AG

^1^Identification level, 1 = Identified compounds; 2 = Putatively annotated compounds; 3 = Putatively characterized compound classes

^2^ Mass to charge ratio m/z.

^3^For stability, “t” represents the number of times that the metabolite was selected in the leave one out procedure (LOO) and *P* the associated probability.

^4^For effect size, “t” represents the absolute value of the regression coefficient of the metabolite *P* the associated probability.

^5^Direction based on the regression coefficient it indicates metabolite concentration higher in AA and AG genotype

### Gene expression analyses—Intestinal mucosa

No significant differences between *FUT1* genotypes were observed in the intestinal expression of analysed genes (**S2 Table**), but the *COX2* expression was higher in mucosa from the mid colon compared to the mucosa from the other gut segments (P = 0.02), and *OCLN* expression was higher (P = 0.008) in mucosa from the ileum compared to mucosa from the caecum and mid colon. A trend of significance (P < 0.1) was observed for *FUT1* genotypes and tissue interaction for *ZO-1* expression.

### Microbiological enumerations by plating and organic acid concentrations

#### Faeces

Higher faecal numbers of haemolytic bacteria at 34 days of age (P = 0.003) and a tendency to higher numbers of lactic acid bacteria (P = 0.06) were observed in *FUT1*^AG^ piglets (**Table 2**). The number of haemolytic bacteria was similar between day 5 and 28, whereas a significant increase was detected on day 34 (P < 0.001). An age-dependent decrease of *Clostridium perfringens* was found with numbers being lowest on day 28 (P < 0.001) and 34 (P < 0.001). The same was observed for lactic acid bacteria, with lower numbers on day 28 (P ≤ 0.03) and day 34 (P ≤ 0.005) compared to day 5 and 7. There was no significant effect of genotype or age on the number of Enterobacteriaceae or total anaerobic bacteria. The interaction between *FUT1* genotype and age showed a trend of significance in the number of Enterobacteriaceae (P < 0.1) and a significant effect in the number of haemolytic bacteria (P = 0.03). Furthermore, no difference in the concentration of faecal short chain fatty acids between genotypes was determined, except for a tendency towards a lower concentration of butyric acid in *FUT1*^AA^ piglets (P < 0.1) (**Table 3**). The concentration of acetic acid was higher at 34 days of age (one-week post-weaning) compared to day 5, 7, 14, and 21 of age (suckling period) (P < 0.05). The concentration of propionic acid was constant between day 5 and 21, but at 34 days of age, a significant increase in propionic acid was observed (P < 0.001). A tendency to a lower concentration of butyric acid (P < 0.01) with age was measured, whereas no effect of age was detected for valeric, iso-butyric and iso-valeric acid (data not shown).

**Table 2:** Enumeration (log cfu/g sample) of selected microbial groups in faeces from piglets at 5, 7, 14, 21, 28 and 34 days of age^1^.

Item	Genotype^2^				*#*	*P*-*value*		
	*FUT1*^AG^		*FUT1*^AA^			G^3^	A^4^	G^3^ x A^4^
Enterobacteriaceae						0.3	0.38	0.08
5	8.5	(7.7–9.2)	8.7	(8.0–9.5)				
7	8.3	(7.5–9.0)	8.6	(7.9–9.3)				
14	8.3	(7.5–9.1)	8.1	(7.4–8.8)				
21	8.2	(7.4–8.9)	8.1	(7.4–8.8)				
28	8.4	(7.7–9.1)	7.9	(7.2–8.6)				
34	8.9	(8.2–9.6)	8	(7.3–8.7)				
Haemolytic bacteria						0.02	< .0001	0.03
5	<6.2^A^ (2)	(5.4–7.1)	<6.3^A^^B^ (1)	(5.2–7.5)				
7	<6.2^A^ (9)	(5.8–6.6)	<6.3^A^ (5)	(5.8–6.8)				
14	<6.3^A^ (3)	(5.7–6.9)	<6.3^A^ (7)	(5.8–6.7)				
21	<6.3^A^ (7)	(5.8–6.7)	<6.2^A^ (6)	(5.8–6.7)				
28	<6.3^A^ (7)	(5.9–6.7)	<6.3^A^ (6)	(5.8–6.7)				
34	8.7^a^^B^	(8.3–9.1)	<7.5^b^^B^ (3)	(7.1–7.9)				
*Clostridium perfringens*						0.6	< .0001	0.97
5	7.7	3.3–12.0	7.5	3.5–11.5	ab			
7	<8.0 (1)	3.2–12.7	7.8	3.5–12.1	b			
14	7.5	3.6–11.4	<7.4 (1)	3.2–11.6	ab			
21	<6.6 (1)	2.2–11.1	6.5	2.2–10.8	a			
28	5.2	0.7–9.6	<5.0 (1)	0.7–9.3	c			
34	<3.0 (6)	0–7.4	<2.8 (3)	0–7.2	d			
Lactic acid bacteria						0.06	0.0002	0.25
5	9.7	9.3–10.0	9.4	9.0–9.8	a			
7	9.6	9.3–10.0	9.4	9.0–9.7	a			
14	8.9	8.5–9.3	8.7	8.3–9.0	b			
21	9.1	8.8–9.4	8.8	8.5–9.2	a			
28	9	8.6–9.3	8.7	8.4–9.0	b			
34	8.8	8.5–9.2	8.6	8.2–8.9	b			
Total anaerobic bacteria						0.86	0.43	0.36
5	9.9	9.7–10.2	9.9	9.7–10.2				
7	9.7	9.4–9.9	9.7	9.4–9.9				
14	9.7	9.5–10.0	9.7	9.5–10.0				
21	9.7	9.5–10.0	9.8	9.5–10.0				
28	9.8	9.6–10.0	9.8	9.6–10.0				
34	9.8	9.5–10.0	9.8	9.6–10.0				

^1^ Values are presented as least square means and 95% confidence intervals (in parentheses).

^2^ Number of piglets: *FUT1*^AG^ = 7, except day 5 (n = 8), day 7 (n = 9), and day 14 (n = 4); *FUT1*^AA^ = 6, except day 5 (n = 4), day 7 (n = 5), and day 14 (n = 7).

^3^ G = Genotype.

^4^ A = Age.

#: Rows with different letters, within a microbial group, are significantly different (P < 0.05).

^A,B^ Values with different superscripts within a column are significantly different (P *<* 0.05).

^a,b^ Values with different superscripts within a row are significantly different(P *<* 0.05).

<: Indicates that at least one of the observations used to calculate the least square mean was below detection level. Numbers in brackets indicate the number of samples below detection level.

**Table 3:** Short-chain fatty acid concentration (mmol/kg sample) in faeces from *FUT1*^AG^ and *FUT1*^AA^ piglets at 5, 7, 14, 21, 28, and 34 days of age^1^.

Item	Genotype^2^				#	*P-value*		
	*FUT1*^*AG*^		*FUT1*^*AA*^			G^3^	A^4^	G^3^ x A^4^
Acetic acid						0.44	< .0001	0.23
5	42.3	(32.0–52.7)	40	(29.5–49.6)	a			
7	38.1	(29.0–47.1)	35	(26.0–44.6)	a			
14	32.7	(22.1–43.4)	30	(20.4–39.4)	a			
21	38	(28.7–47.4)	35	(26.2–44.2)	a			
28	46.5	(37.1–55.9)	44	(34.7–52.7)	ab			
34	58.9	(49.9–67.9)	56	(47.2–65.0)	b			
Propionic acid						0.19	< .0001	0.13
5	12.4	(8.1–16.7)	10	(6.2–14.6)	a			
7	10.9	(7.2–14.6)	8.9	(5.1–12.8)	a			
14	12.3	(7.8–18.8)	10	(6.4–14.2)	a			
21	12.6	(8.8–16.4)	11	(6.9–14.3)	a			
28	12	(8.2–15.9)	10	(6.4–13.7)	a			
34	22.4	(18.7–26.0)	20	(16.7–24.0)	b			
Butyric acid						0.09	0.09	0.48
5	6.6	(3.5–10.7)	4.6	(2.2–8.0)				
7	5.3	(2.9–8.4)	3.5	(1.6–6.2)				
14	3	(1.0–6.1)	1.7	(0.5–3.8)				
21	2.5	(0.9–4.8)	1.3	(0.3–3.1)				
28	4	(1.9–6.9)	2.5	(1.0–4.7)				
34	5.3	(3.0–8.4)	3.6	(1.7–6.1)				
A+P+B^5^						0.23	< .0002	0.14
5	61.7	(46.5–77.0)	55	(40.6–70.1)	a			
7	56	(43.0–69.0)	50	(36.1–63.2)	a			
14	49.4	(33.5–65.2)	43	(29.2–56.8)	a			
21	54.3	(40.8–67.8)	48	(35.0–61.0)	a			
28	63.8	(50.3–77.3)	57	(44.4–70.4)	ab			
34	87.1	(74.3–100.0)	81	(67.9–93.5)	b			

^1^ Values are presented as least square means and 95% confidence intervals (in parentheses).

^2^. Number of piglets: *FUT1*^AG^ = 10, except, day 5 (n = 8), day 7 (n = 9) and day 14 (n = 7); *FUT1*^AA^ = 6, except day 5 (n = 4), day 7 (n = 5) and day 14 (n = 7).

^3^ G = *FUT1* Genotype.

^4^ A = Age.

^5^ A+P+B = acetic + propionic + butyric acid.

#: Rows with different letters, within a short-chain fatty acid group, are significantly different (P < 0.05).

#### Digesta

Genotype did not influence dry matter content or pH (**S3 Table**) of gastrointestinal digesta. *FUT1*^AG^ piglets had a higher number of Enterobacteriaceae (P ≤ 0.02) in digesta from the distal small intestine, caecum, and mid colon; of haemolytic bacteria (P = 0.02) in the stomach, small intestine, caecum, and colon; and of total anaerobic bacteria in the distal small intestine compared to *FUT1*^AA^ piglets (P = 0.004) (**Table 4**). The concentration of acetic acid was higher in the mid colon of *FUT1*^AG^ piglets (P = 0.01) (**Table 5**). Digesta concentrations of propionic, butyric, lactic, valeric, and the sum of iso-butyric and iso-valeric acid were similar in both genotypes.

**Table 4:** Enumeration (log cfu/g sample) of selected microbial groups in digesta from the gastrointestinal tract of 34 days old piglets (one week post-weaning)^1^ from the *FUT1*^AG^ and *FUT1*^AA^ groups.

Item	Genotype^2^				*P-value*		
	*FUT1*^AG^		*FUT1*^AA^		G^3^	S^4^	G^3^ x S^4^
Enterobacteriaceae					0.002	< .0001	0.004
Stomach	5.6	(4.8–6.5)	5.9	(5.0–6.7)			
Distal small intestine	8.8^a^	(7.9–9.7)	7.5^b^	(6.6–8.3)			
Caecum	8.8^a^	(8.0–9.7)	7.8^b^	(7.0–8.7)			
Mid colon	8.9^a^	(8.0–9.8)	7.9^b^	(7.1–8.8)			
Haemolytic bacteria					0.02	< .0001	0.28
Stomach	5.2	(4.5–6.0)	<4.4 (1)	(3.8–5.1)			
Distal small intestine	7.9	(6.8–9.2)	<6.8 (4)	(5.8–7.9)			
Caecum	8.6	(7.4–10)	<7.3 (4)	(6.3–8.5)			
Mid colon	8.6	(7.4–10)	<7.3 (3)	(6.3–8.5)			
*Clostridium perfringens*					0.93	0.63	0.24
Stomach	<3.2 (1)	(2.6–3.7)	3.2	(2.6–3.7)			
Distal small intestine	<2.8 (5)	(2.3–3.4)	<2.8 (4)	(2.3–3.4)			
Caecum	<3.0 (4)	(2.5–3.6)	<3.0 (4)	(2.5–3.6)			
Mid colon	<3.0 (4)	(2.4–3.5)	<3.0 (4)	(2.4–3.5)			
Lactic acid bacteria					0.29	< .0001	0.4
Stomach	9	(8.4–9.6)	8.7	(8.1–9.3)			
Distal small intestine	8.3	(7.8–8.9)	8	(7.3–8.6)			
Caecum	8.6	(8.0–9.2)	8.3	(7.6–8.8)			
Mid colon	8.6	(8.0–9.2)	8.3	(7.7–8.9)			
Total anaerobic bacteria					0.02	< .0001	0.01
Stomach	9	(8.7–9.4)	9.3	(9.0–9.6)			
Distal small intestine	8.9a	(8.6–9.3)	8.3b	(8.0–8.6)			
Caecum	9.3	(9.0–9.7)	9.2	(8.9–9.5)			
Mid colon	9.4	(9.1–9.7)	9.4	(9.1–9.7)			

^1^ Samples from the stomach, distal small intestine, caecum and mid colon were analysed. Values are presented as least square means and 95% confidence intervals (in brackets).

^2^ Number of piglets: *FUT1*^AG^ n = 10; *FUT1*^AA^ n = 7.

^3^ G = *FUT1* Genotype.

^4^ S = Intestinal segment.

^a,b^ Values with different superscripts within a row are significantly different (*P <* 0.05).

<: Indicates that at least one of the observations used to calculate the least square mean was below detection level, followed by numbers in brackets indicating the number of samples below detection levels.

**Table 5:** Organic acid concentrations in digesta (mmol/kg wet sample) from the gastrointestinal tract of 34 days old piglets (one week post-weaning) belong to *FUT1*^AG^ and *FUT1*^AA^ groups.

Item^1^	Genotype^2^				*P-value*		
	*FUT1*^*AG*^		*FUT1*^*AA*^		G^3^	S^4^	G^3^ x S^4^
Lactic acid^5^					1	< .0001	0.9
Stomach	19.6	(5.3–62.7)	19.5	(6.9–50.2)			
Distal small intestine	5.6	(0.6–20.4)	5.5	(1.1–16.2)			
Acetic acid					0.01	< .0001	0.01
Stomach	21.1	(4.2–37.9)	26.8	(11.6–41.9)			
Distal small intestine	4.2	(0.0–21.0)	5.4	(0.0–20.6)			
Caecum	47.6	(30.7–64.4)	39	(24.1–53.9)			
Mid colon	54.5^a^	(37.7–71.4)	39.4^b^	(24.4–54.4)			
Propionic acid^6^					0.02	0.0001	0.01
Stomach	8.1	(2.7–13.6)	11.7	(6.9–16.5)			
Caecum	16.7	(11.3–22.2)	13.2	(8.1–18.3)			
Mid colon	16.6	(11.1–22.0)	11.9	(6.9–16.9)			
Butyric acid^7^					0.9	< .0001	0.8
Stomach	8.3	(1.4–48.0)	8.1	(0.5–41.2)			
Caecum	2.3	(6.1–30.3)	2.2	(3.8–24.0)			
Mid colon	1.9	(6.8–29.0)	1.8	(4.6–23.6)			

^1^ Samples from the stomach, distal small intestine, caecum and mid colon were analysed. Values are presented as least square means and 95% confidence interval (in parentheses).

^2^ Number of piglets: *FUT1*^AG^ n = 10, except the distal small intestine (n = 9); *FUT1*^AA^.n = 7, except the mid colon (n = 6) and caecum (n = 5).

^3^ G = *FUT1* Genotype.

^4^ S = Intestinal segment.

^5^ Samples from the caecum and mid colon had values below detection level.

^6^ Samples from the distal small intestine had values below detection level. No superscripts as the G x S interaction was non-significant after pairwise comparisons using Bonferroni correction.

^7^Samples from the distal small intestine had values below detection level.

^a,b^ Values with different superscripts within a row are significantly different (P *<* 0.05).

### Quantification of E. coli attachment to distal small intestinal tissue

The *E*. *coli* numbers in the small intestinal tissue were similar in both genotypes (P = 0.41). However, *FUT1*^AG^ piglets had numerically higher *E*. *coli* numbers before (*FUT1*^AG^ 6.3 log cfu/g *versus FUT1*^AA^ 6.0 log cfu/g), and after *E*. *coli* O138:F18 inoculation (*FUT1*^AG^ 8.1 log cfu/g *versus FUT1*^AA^ 7.8 log cfu/g) than the *FUT1*^AA^ piglets.

## Discussion

In this study, a metabolomics approach was applied to evaluate the influence of *FUT1* genotype on system-biological responses in healthy piglets during the suckling and post-weaning period. Bacterial counts for important bacteria groups involved in the microbial balance such as lactic acid bacteria, Enterobacteriaceae and haemolytic bacteria, gene expression analysis for genes associated with intestinal epithelial barrier (ZO-1 and OCL) [33] and mucosal gut inflammation such as the TNF-α, and COX-2, which are upregulated upon antigens stimulation, and IL-10, which encodes for an anti-inflammatory cytokine that plays a key role in immune response [34], were analysed to further add information on the impact of genotype on the host’s gastrointestinal homeostatic environment.

Age of the piglets influenced the plasma metabolomics profile and faecal bacterial enumerations, which is ascribed to the piglets physiological and immunological maturation during the initial phase of life [35,36], and to the dietary change that occurred at weaning. In our study, we found a progressive decrease in the number of lactic acid bacteria and *Clostridium perfringens* in the period from 5 to 34 days of age. Lactic acid bacteria, especially *Lactobacillus spp*., are important in maintaining intestinal homeostasis as they are capable of reducing pH by lactic acid production, compete with potential pathogens, and produce bacteriocins [37,38]. However, the number of *Lactobacillus* has been observed to decrease with the age and specially after the weaning transition period [39–42]. The process of weaning the piglet from the sow and thereby the switch from milk to solid feed significantly increased in the number of haemolytic bacteria in faeces between 28 and 34 days of age. Indeed, the gastrointestinal microbiota is strongly influenced by diet, both by the change in milk composition during suckling period [39–42], by the switch from the dam’s milk to the solid feed, and generally an increase in microbial richness and complexity occurs between birth and the post-weaning period [40,41]. The observed effect of age of piglets on the metabolomics profile and bacterial population will not be discussed in depth here, as it was not the primary focus of the present study. However, it is important to note that an interaction between *FUT1* genotype and age was detected in the number of haemolytic bacteria in the faeces on day 34, suggesting that the interaction of factors can affect the number of possible pathogenic bacteria.

The presented metabolomics results shed light on the influence of *FUT1* genotype to specific architecture in the pig metabolome. The PCA score plot showed relatively consistent clusters attributable to *FUT1* genotype when considering each of the single time points separately, i.e., day 7, 28, and 34. The influence of genotype was clearest in the metabolome profile of young animals (day 7 and 28) compared to the plasma metabolic profile after weaning (day 34). In general, no particular pathway in the plasma metabolome was shown to be responsible for the differentiation of the two *FUT1* genotypes. Nevertheless, discriminant compounds were detected and validated thought the LOO procedure in the metabolomics data from day 7, 28, and 34. On day 7, the *FUT1*^AG^ piglets had higher plasma values of metabolites originating from the metabolism of phenylalanine, hippuric acid, tryptophan, and oxindole. Hippuric acid, constituted by the conjugation of benzoic and acyl glycine, is synthesised in the intestine, liver, and spleen [43], and it accumulates in the blood, causes ammoniagenesis stimulation, and it is generally associated with an increased feed consumption of phenylalanin or phenolic compounds, and with the activity of the gastrointestinal microbiota [44–46]. We speculate that the higher hippuric acid amount in *FUT1*^AG^ piglets could be ascribed to their higher feed intake since the sMLPLS-DA was performed on the metabolomics data corrected for BW of the animals on day 7, and further that the diet (i.e. sow milk and post weaning diet) was not different between *FUT1*^AG^ and *FUT1*^AA^ pigs. However, the microbial activity in the gut is also associated with the concentration of hippuric acid in the blood [45], and in humans lower urinary hippurate levels in Crohn’s disease patients have been associated with an altered gut microbial metabolism [46]. No significant difference in the number of various microbial groups in faeces of the two groups was found on day 7, though the analysis of digesta at this age might have provided different results. The oxindole, also named 1,3-Dihydroindol-2-one (KEEG C12312), is a putative tryptophan metabolite, which is involved in the benzoxazinoid biosynthesis, it is a secondary metabolite that serves as an important factor in the host’s resistance against microbial pathogens. No further information has been found regarding this compound in porcine biological systems. On the other hand, the animals with the *FUT1*^AA^ genotype showed a higher intensity in the signal of betaine, a small N-trimethylated amino acid that originates from the oxidation of choline, which functions as a methyl donor. Donation of methyl groups is important for an optimal liver function, cellular replication, and detoxification reactions [47]. Furthermore, a decreased betaine level has been associated with stress due to weaning and exposure to *E*. *coli* [48]. Our results identified two nitrogenous bases, guanosine and 3-methylguanine level, at weaning (day 28) that were affected by genotype, *FUT1*^AA^ *versus FUT1*^AG^. The higher level of guanosine in *FUT1*^AG^ piglets probably indicated that these animals could had suffered of an inflammatory process. Indeed, it has been shown that extracellular guanosine tended to increase and accumulate in response to injury as oxygene and glucose deprivation in rats [49,50] and it promotes a glioprotective mechanism against the inflammatory and oxidative damage induced by lipopolysaccharide (LPS) exposure in primary cultures of hippocampal astrocytes by the activation of heme oxygenase-1 (HO-1) pathway [51]. Piglets with the *FUT1*^AA^ genotype were characterized by a higher 3-methylguanine level, a metabolite involved in the DNA repair process [52]. However, we are not aware of any animal studies available on the role of this compound in plasma, and our work does therefore not allow us to associate this metabolite with animal health or biological processes.

Regarding the post-weaning period, the metabolome profile of plasma samples obtained on day 34 showed that the influence of *FUT1* genotype on the plasma metabolome had decreased when compared to day 7 and 28. This result may be ascribed to a range of different factors perturbing the piglet’s physiological homeostasis after weaning. The effect of *FUT1* genotype on the metabolic profile discriminations was attributed to compounds such as tauroursodeoxycholic acid, a bile acid compound, and L-phenylalanine, an amino acid precursor. Phenylalanine is an essential amino acid, derived by the protein catabolism and which is mainly converted to tyrosine in the kidneys [53], while tauroursodeoxycholic acid is involved in the digestion and intestinal absorption of hydrophobic nutrients. A higher level of these compounds was found in plasma of in plasma of *FUT1*^AA^ piglets, and may hence be relevant for their nutrient absorption capability. On the other hand, in our study, *FUT1*^AG^ piglets have been found to have a higher level of genistein 5-o-glucuronide, a polyphenol metabolite involved in the intestinal barrier regulation [54]. Our results on the expression of candidate genes involved in the intestinal barrier function (*ZO-1* and *OCLN*) and immunity response (*IL10*, *TNF*-α and *COX*-2) showed no differences between *FUT1*^AA^ and *FUT1*^AA^ piglets, however, it could be possible that the differences due to *FUT1* variants were not sufficient to demonstrate differences in these gene markers that are generally stimulated after infection, indeed our animals stayed healthy until the end of the trial.

In order to assess the microbiota metabolism along the gastrointestinal tract of the two *FUT1* groups, organic acid analysis was carried out. Acetic acid, which is the major short-chain fatty acid in the gut, was found in higher concentrations in mid-colon digesta of *FUT1*^AG^. As the short-chain fatty acid profile reflects bacterial metabolism [55], the higher colonic concentration of acetic acid in *FUT1*^AG^ piglets most likely reflects bacterial community differences between genotypes. Bacterial culture showed a higher number of haemolytic bacteria, both in faeces and digesta, and of Enterobacteriaceae in digesta content from *FUT1*^AG^ piglets. *E*. *coli* belongs to this family and has been shown to produce higher amounts of acetic acid at high growth rates [56]. This alone is unlikely to explain the difference in acetic acid concentrations as we have a lack of information about the detailed commensal bacteria present in the intestinal tract of the animals, but all these results together may indicate that *FUT1* genotype can influence the microbiota balance, the microbial metabolism and thus the host physiological condition.

We were not able to detect significantly higher *E*. *coli* F18 numbers in the small intestinal tissue using an *ex vivo* porcine intestinal model. Further, we were not able to detect weight gain differences, although some plasma metabolomic differences between genotypes could be ascribed to feed intake. The lack of significance between the genotypes regarding epithelial adherence of *E*. *coli* as obtained in the *ex vivo* challenge model, and the pig body weight responses might be due to the low number of replicates in the present study.

## Conclusion

The results obtained in the present study contribute to the existing knowledge on *FUT1* polymorphisms effect in pigs immune responses and gastrointestinal bacteria. Our investigation showed that *FUT1* genotype influenced the number of haemolytic bacteria and Enterobacteriaceae in the intestinal content. In addition, *FUT1* genotype influenced the plasma metabolic profile, especially during the pre-weaning period, and specific plasma metabolites related with gut microbial metabolism, inflammation and nutrient absorption capability. Further study on host *FUT1* genotype are desirable to improve the knowledge on the relationship between *FUT1* genotype, the mucosal bacterial community, and the host immune response in order to elucidate the perspective of e.g. breeding strategies on gut health parameters others than diarrhoea. Moreover, our work encourages future studies that evaluate the role and contribution of Fucosyltransferase genes family on the mechanisms involved in the pathophysiology of intestinal disease and inflammation.

## Supporting information

S1 Table Complete list of discriminant plasma metabolites for *FUT1*^AG^ and *FUT1*^AA^ obtained from piglets at 7, 28 and 34 days of age by the sMLPLS-DA. (DOCX)

S2 Table Relative expression of selected genes in mucosa from the stomach, ileum, caecum and mid colon of piglets 34 days of age (one-week post-weaning) belong to *FUT1*^AG^ and *FUT1*^AA^ groups. (DOCX)

S3 Table Dry matter content (%) and pH of digesta from the gastrointestinal tract of 34 days old piglets (one-week post-weaning)^1^. (DOCX)
