# Supplementary material for: Effects of alpha-(1,2)-fucosyltransferase genotype variants on plasma metabolome, immune responses and gastrointestinal bacterial enumeration of pigs pre- and post-weaning
Source: PLoS One. 2018 Aug 27;13(8):e0202970. doi: 10.1371/journal.pone.0202970 (PMC6110508; doi:10.1371/journal.pone.0202970)
Supplement: S1 Table — (DOCX) [file pone.0202970.s001.docx]

**Table S1. Title: Complete list of discriminant plasma metabolites for *FUT1*^AG^ and *FUT1*^AA^ obtained from piglets at 7, 28 and 34 days of age by the sMLPLS-DA**

| Metabolite | Charge | Retention time (min) | m/z^1^ | Genotype | | | | Stability^2^ | | Effect size^3^ | | Direction^4^ | |
| --- | --- | --- | --- | --- | --- | --- | --- | --- | --- | --- | --- | --- | --- |
|  |  |  |  | *FUT1*^AG^ | | *FUT1*^AA^ | |  |  |  |  |  |  |
|  |  |  |  | Mean | ds | Mean | ds | t | P | t | P |  |  |
| Day 7 | | | | | | | | | | | | | |
| [M+Na] Betaine | POS | 0.63 | 140.9 | 6794 | 195.3 | 6590 | 207 | 6 | 0.08 | -0.04 | 0.03 | AA | |
| Unidentified | POS | 0.63 | 196.87 | 31470 | 1455.5 | 28593 | 1388.7 | 8 | 0.04 | -0.05 | 0.02 | AA | |
| Unidentified | POS | 0.92 | 315.02 | 3574 | 141.6 | 2714 | 382 | 6 | 0.07 | -0.04 | 0.03 | AA | |
| Unidentified | POS | 1.9 | 255.15 | 14726 | 2541.1 | 6506 | 1627.7 | 9 | 0.02 | -0.05 | 0.02 | AA | |
| N-methyl-2-pyrrolidinone | POS | 1.91 | 102.08 | 7242 | 237.3 | 8167 | 160.9 | 10 | 0.01 | 0.05 | 0.01 | AG | |
| Hippuric acid | POS | 3.26 | 180.07 | 4546 | 1733.8 | 13273 | 2987.9 | 6 | 0.08 | 0.04 | 0.01 | AG | |
| Oxindole | POS | 3.37 | 134.06 | 9476 | 840.5 | 12923 | 2446.2 | 6 | 0.07 | 0.04 | 0.02 | AG | |
| Unidentified | POS | 6.36 | 355.26 | 15127 | 507.3 | 17922 | 526 | 8 | 0.04 | 0.05 | 0.02 | AG | |
| Unidentified | POS | 6.47 | 420.2 | 1069 | 75.8 | 1325 | 26.7 | 10 | 0.02 | 0.05 | 0.01 | AG | |
| 3-Oxo-5beta-chol-7-en-24-oic Acid | POS | 7.28 | 374.28 | 8140 | 440 | 9412 | 288.2 | 6 | 0.07 | 0.04 | 0.02 | AG | |
| Unidentified | POS | 7.49 | 451.33 | 7938 | 1241.1 | 13799 | 326.9 | 9 | 0.03 | 0.05 | 0.02 | AG | |
| Unidentified | POS | 7.91 | 711.45 | 1477 | 493.6 | 2190 | 43.1 | 6 | 0.09 | 0.04 | 0.02 | AG | |
| Unidentified | POS | 8.59 | 335.22 | 8297 | 4572.7 | 14600 | 1420 | 6 | 0.06 | 0.04 | 0.02 | AG | |
| Adduct of 496.3397 (PC(16:0/0:0)[U]) | POS | 9.29 | 524.81 | 3901 | 252.2 | 4195 | 320.5 | 7 | 0.04 | 0.04 | 0.01 | AG | |
| Unidentified | POS | 10.48 | 468.44 | 9277 | 1245.1 | 13512 | 922 | 9 | 0.02 | 0.05 | 0.02 | AG | |
| Unidentified | POS | 10.55 | 580.32 | 3660 | 108.6 | 4670 | 292.6 | 7 | 0.05 | 0.05 | 0.02 | AG | |
| [M+H-H2O]+ PS(13:0/14:1(9Z)) | POS | 10.67 | 646.41 | 1561 | 935.9 | 9513 | 187.4 | 11 | 0.01 | 0.05 | 0.01 | AG | |
| Adduct of 524.3718 PC(O-16:0/2:0)[U] | POS | 10.74 | 562.45 | 9618 | 285.5 | 4253 | 1012 | 9 | 0.02 | -0.05 | 0.01 | AA | |
| Unidentified | POS | 11.13 | 315.18 | 8206 | 243.6 | 3363 | 647.1 | 10 | 0.02 | -0.05 | 0.01 | AA | |
| Unidentified | POS | 12.56 | 790.56 | 8778 | 338.5 | 12631 | 1269.6 | 9 | 0.02 | 0.05 | 0.01 | AG | |
| Unidentified | POS | 12.59 | 228.2 | 5467 | 363.1 | 4093 | 808.9 | 5 | 0.09 | -0.04 | 0.01 | AA | |
| Day 28 | | | | | | | | | | | | | |
| 3-Methylguanine | POS | 0.91 | 166.07 | 7044 | 552.9 | 5824 | 241.9 | 15 | 0 | 0.46 | 0 | AA | |
| Guanosine | POS | 1.27 | 284.1 | 4533 | 1645.9 | 7980 | 1016.3 | 12 | 0.02 | 0.42 | 0 | AG | |
| Unidentified | POS | 2.61 | 283.18 | 10636 | 562.8 | 9164 | 482.1 | 14 | 0.01 | 0.45 | 0 | AA | |
| Unidentified | NEG | 9.81 | 606.33 | 1839 | 357.4 | 1287 | 232.4 | 14 | 0.01 | 0.44 | 0 | AA | |
| Day 34 | | | | | | | | | | | | | |
| Sulfuric acid, 1,2-Dichloroethane, 1,1-Dichloroethane | NEG | 0.91 | 96.96 | 77755 | 18060.2 | 67337 | 8471.2 | 8 | 0.07 | -0.04 | 0.02 | AA | |
| (S)-2-Hydroxyglutarate | NEG | 1.9 | 147.05 | 17737 | 1058.5 | 15301 | 796.7 | 15 | 0 | -0.06 | 0.02 | AA | |
| L-Phenylalanine | NEG | 1.9 | 164.07 | 135175 | 8329.9 | 122786 | 6632 | 8 | 0.07 | -0.04 | 0.04 | AA | |
| Unidentified | POS | 2.01 | 217.11 | 6712 | 1416.9 | 5296 | 678.5 | 15 | 0 | -0.06 | 0.02 | AA | |
| Ile Glu Phe Gly | POS | 3.89 | 465.24 | 5588 | 1009.7 | 5903 | 2107.8 | 14 | 0.01 | 0.05 | 0.04 | AG | |
| Genistein 5-O-glucuronide | NEG | 4.18 | 445.08 | 3886 | 1322.5 | 6445 | 2268.6 | 8 | 0.07 | 0.04 | 0.02 | AG | |
| Unidentified | POS | 5.03 | 531.32 | 56924 | 2439.6 | 63111 | 6839 | 8 | 0.07 | 0.04 | 0.03 | AG | |
| Unknown | POS | 5.42 | 900.56 | 8011 | 1101.7 | 9215 | 780.5 | 7 | 0.09 | 0.04 | 0.03 | AG | |
| Tauroursodeoxycholic acid | NEG | 5.62 | 498.29 | 73051 | 80247.3 | 21852 | 26902.9 | 7 | 0.08 | -0.04 | 0.03 | AA | |
| Tauroursodeoxycholic acid | POS | 5.65 | 500.31 | 14039 | 15505.8 | 7702 | 4255.8 | 8 | 0.06 | -0.04 | 0.02 | AA | |
| Unknown | POS | 6.48 | 359.15 | 53343 | 3569.9 | 48571 | 6000.3 | 7 | 0.09 | -0.04 | 0.04 | AA | |
| Unknown | POS | 6.48 | 499.26 | 5535 | 354.3 | 5135 | 371.2 | 9 | 0.05 | -0.05 | 0.04 | AA | |
| Unknown | NEG | 6.5 | 403.14 | 4849 | 271.5 | 3977 | 409.2 | 14 | 0.01 | -0.06 | 0.05 | AA | |
| Unknown | POS | 7.39 | 378.24 | 3092 | 523.3 | 2713 | 431.3 | 9 | 0.05 | -0.05 | 0.02 | AA | |
| Fragment of 293.175 | NEG | 7.68 | 221.15 | 9943 | 2731.6 | 5201 | 1447.5 | 9 | 0.06 | -0.05 | 0.03 | AA | |
| Fragment of 293.175 | NEG | 7.68 | 236.11 | 16053 | 4396.8 | 8006 | 3120.3 | 7 | 0.1 | -0.04 | 0.04 | AA | |
| Unidentified | NEG | 7.68 | 293.18 | 66182 | 16981.6 | 33551 | 11803.2 | 9 | 0.05 | -0.05 | 0.03 | AA | |
| Unknown PC/LysoPC | POS | 8.9 | 560.3 | 1750 | 196.9 | 1436 | 106.7 | 15 | 0 | -0.06 | 0.03 | AA | |
| PC/LysoPC or fragment | POS | 9.54 | 401.31 | 3196 | 312.8 | 2903 | 291.7 | 7 | 0.09 | -0.04 | 0.06 | AA | |
| Unknown | NEG | 9.59 | 480.35 | 20622 | 4346.6 | 17210 | 5472.3 | 9 | 0.04 | -0.05 | 0.02 | AA | |
| PC(18:0/0:0) | NEG | 10.45 | 560.33 | 4109 | 2918.4 | 7489 | 7674.1 | 11 | 0.03 | 0.05 | 0.04 | AG | |
| Unidentified | POS | 11.71 | 526.43 | 14734 | 663 | 15772 | 555.7 | 7 | 0.09 | -0.04 | 0.05 | AA | |
| Unknown | POS | 12.34 | 531.39 | 5910 | 1151.7 | 5352 | 493.8 | 7 | 0.08 | -0.04 | 0.04 | AA | |
| Fragment of 226.181 | POS | 12.59 | 227.17 | 23453 | 4909.5 | 18057 | 4780.5 | 10 | 0.04 | -0.05 | 0.04 | AA | |
| Unknown | POS | 12.6 | 226.18 | 13559 | 3358.2 | 11282 | 2751.5 | 12 | 0.02 | -0.05 | 0.02 | AA | |
| Fragment of 226.181 | POS | 12.6 | 228.2 | 5641 | 1899.8 | 4966 | 1407.2 | 6 | 0.09 | -0.04 | 0.03 | AA | |

^1^ Mass to charge ratio m/z.

^2^For the stability “t” represents the number of times that the metabolite was selected in the leave one out procedure (LOO) and *P* the associated probability.

^3^For the effect size “t” represents the absolute value of the regression coefficient of the metabolite *P* the associated probability.

^4^Direction: based on the regression coefficient it indicates metabolite concentration higher in AA and AG genotype.
