# Supplementary material for: Effects of alpha-(1,2)-fucosyltransferase genotype variants on plasma metabolome, immune responses and gastrointestinal bacterial enumeration of pigs pre- and post-weaning
Source: PLoS One. 2018 Aug 27;13(8):e0202970. doi: 10.1371/journal.pone.0202970 (PMC6110508; doi:10.1371/journal.pone.0202970)
Supplement: S2 Table — (DOCX) [file pone.0202970.s002.docx]

**Supplementary Table 2. Relative expression of selected genes in mucosa from the stomach, ileum, caecum and mid colon of piglets 34 days of age (one-week post-weaning) belong to *FUT1*^AG^ and *FUT1*^AA^ groups.**

| Item^1^ | Genotype^2^ | | | | # | *P-value* | | |
| --- | --- | --- | --- | --- | --- | --- | --- | --- |
|  | *FUT1^AG^* | | *FUT1^AA^* | |  |  |  |  |
|  | Mean | SEM | Mean | SEM |  | G^3^ | T^4^ | G^3^xT^4^ |
| *IL10* | | | | | | 0.3 | 0.11 | 0.8 |
| Stomach | 0.1 | 0.02 | 0.15 | 0.03 |  |  |  |  |
| Ileum | 0.14 | 0.02 | 0.15 | 0.03 |  |  |  |  |
| Caecum | 0.14 | 0.02 | 0.18 | 0.02 |  |  |  |  |
| Mid colon | 0.17 | 0.02 | 0.18 | 0.03 |  |  |  |  |
| *COX 2* | | | | | | 0.4 | 0.02 | 0.18 |
| Stomach | 0.03 | 0.02 | 0.03 | 0.01 | a |  |  |  |
| Ileum | 0.02 | 0.01 | 0.02 | 0.01 | a |  |  |  |
| Caecum | 0.02 | 0.01 | 0.03 | 0.01 | a |  |  |  |
| Mid colon | 0.03 | 0.01 | 0.08 | 0.02 | b |  |  |  |
| *TNF-α* | | | | | | 0.22 | 0.14 | 0.41 |
| Stomach | 3.19 | 0.69 | 3.65 | 0.89 |  |  |  |  |
| Ileum | 3.13 | 0.73 | 3.58 | 0.98 |  |  |  |  |
| Caecum | 2.51 | 0.73 | 2.56 | 0.89 |  |  |  |  |
| Mid colon | 2.99 | 0.69 | 5.27 | 0.89 |  |  |  |  |
| *ZO-1* | | | | | | 0.13 | 0.52 | 0.07 |
| Ileum | 1.58 | 0.15 | 2.01 | 0.2 |  |  |  |  |
| Caecum | 1.78 | 0.16 | 1.41 | 0.22 |  |  |  |  |
| Mid colon | 1.71 | 0.15 | 1.69 | 0.19 |  |  |  |  |
| *OCLN* | | | | | | 0.48 | 0.008 | 0.26 |
| Ileum | 0.73 | 0.07 | 0.72 | 0.09 | b |  |  |  |
| Caecum | 0.45 | 0.07 | 0.57 | 0.09 | a |  |  |  |
| Mid colon | 0.63 | 0.07 | 0.53 | 0.09 | a |  |  |  |

^1^ Item*: IL10*, Interleukin 10; *COX-2*, cyclo-oxygenase-2: *TNF-α*, Tumor Necrosis Factor Alfa; *ZO-1*, Zona Occludens 1; *OCLN*, Occludin.

^2^ Number of piglets: *FUT1*^AG^ =10; *FUT1*^AA^ =7.

^3^ G = Genotype.

^4^ T =Tissue.

a,b = Values with different superscripts within a gene are significantly different (*P-value* < 0.05).
