# Supplementary material for: Effects of alpha-(1,2)-fucosyltransferase genotype variants on plasma metabolome, immune responses and gastrointestinal bacterial enumeration of pigs pre- and post-weaning
Source: PLoS One. 2018 Aug 27;13(8):e0202970. doi: 10.1371/journal.pone.0202970 (PMC6110508; doi:10.1371/journal.pone.0202970)
Supplement: S3 Table — (DOCX) [file pone.0202970.s003.docx]

**Table S3 Table. Dry matter content (%) and pH of digesta from the gastrointestinal tract of 34 days old piglets (one week post-weaning)^1^**

| Item | Genotype^2^ | | | | *P*- value | | |
| --- | --- | --- | --- | --- | --- | --- | --- |
|  | *FUT1*^AG^ | | *FUT1*^AA^ | | G^3^ | S^4^ | G^3^xS^4^ |
| Dry matter | | | | | 0.33 | <.0001 | 0.34 |
| Stomach | 25.1 | (20.8-29.5) | 24 | (20.1-27.9) |  |  |  |
| Distal small intestine | 6 | (1.7-10.3) | 4.9 | (1.0-8.7) |  |  |  |
| Caecum | 7.1 | (3.2-10.9) | 5.9 | (2.2-9.6) |  |  |  |
| Mid colon | 7.8 | (3.6-11.9) | 6.6 | (2.9-10.4) |  |  |  |
| pH | | | | | 0.86 | <.0001 | 0.55 |
| Stomach | 3 | (2.7-3.2) | 3 | (2.7-3.2) |  |  |  |
| Distal small intestine | 7.3 | (7.0-7.6) | 7.3 | (7.0-7.5) |  |  |  |
| Caecum | 6.7 | (6.4-6.9) | 6.7 | (6.4-6.9) |  |  |  |
| Mid colon | 6.9 | (6.7-7.2) | 6.9 | (6.7-7.2) |  |  |  |

^1^ Samples from the stomach, distal small intestine, caecum and mid colon were analysed. Values are presented as least square means and 95% confidence intervals (in parentheses).

^2^ Number of piglets: *FUT1*^AG^ =10; *FUT1*^AA^ =7, except: pH *FUT1*^AG^ distal small intestine (n=9); dry matter *FUT1*^AG^ piglets caecum and mid colon (n=5) and *FUT1*^AA^ caecum (n=6).

^3^ G = Genotype.

^4^ S = Intestinal segment.
